# Prevalence and WGS-based characteristics of *Staphylococcus aureus* in the nasal mucosa and pastern of horses with equine pastern dermatitis

**DOI:** 10.1186/s12917-021-03053-y

**Published:** 2022-02-24

**Authors:** Sarah Kaiser-Thom, Vinzenz Gerber, Alexandra Collaud, Joel Hurni, Vincent Perreten

**Affiliations:** 1 Department of Clinical Veterinary Medicine, Vetsuisse Faculty, Swiss Institute of Equine Medicine (ISME), University of Bern, and Agroscope, Bern, Switzerland; 2 Institute of Veterinary Bacteriology, Vetsuisse Faculty, University of Bern, Bern, Switzerland

**Keywords:** Equine pastern dermatitis, Dermatology, *Staphylococcus aureus*, Frequency, Antimicrobial resistance, Virulence factors, WGS, Genotyping

## Abstract

**Background:**

Many contributing factors are involved in the development of equine pastern dermatitis (EPD). Among the most frequently suspected is *Staphylococcus aureus*, known for its pathogenic potential in skin and soft tissue infections. We therefore investigated the association between *S. aureus* carriage and EPD.

**Results:**

One hundred five EPD-affected horses and 95 unaffected controls were examined for the presence of methicillin-resistant and -susceptible *Staphylococcus aureus* (MRSA and MSSA) on the pastern skin and in the nostrils. *S. aureus* isolates were cultivated from swab samples on selective MSSA and MRSA chromogenic agar and identified using MALDI-TOF MS. Isolates were analysed by Illumina whole genome sequencing for genetic relatedness (cgMLST, *spa* typing), and for the presence of antimicrobial resistance and virulence determinants. A markedly higher proportion of samples from EPD-affected horses proved positive for *S. aureus*, both from the pastern (59.0 % vs. 6.3 % in unaffected horses; *P*<0.001), and from the nose (59.0 % vs. 8.4 %; *P*<0.001). Isolates belonged to 20 sequence types (ST) with lineages ST15-t084 (*spa*) (18 %), ST1-t127 (13 %), and ST1-t1508 (12 %) being predominant. Eight *S. aureus* were MRSA ST398-t011 and ST6239-t1456, and contained the staphylococcal cassette chromosome SCC*mec*IVa. Antimicrobial resistance genes were almost equally frequent in pastern and in nasal samples, whereas some virulence factors such as the beta-hemolysin, ESAT-6 secretion system, and some enterotoxins were more abundant in isolates from pastern samples, possibly enhancing their pathogenic potential.

**Conclusions:**

The markedly higher prevalence of *S. aureus* containing specific virulence factors in affected skin suggests their contribution in the development and course of EPD.

**Supplementary Information:**

The online version contains supplementary material available at 10.1186/s12917-021-03053-y.

## Background

Equine pastern dermatitis (EPD) is one of the most frequently encountered skin disorders in equine practice. It is considered a syndrome rather than a disease entity and can result in a range of clinical signs, most typically including erythema, alopecia, scales, crusts, and thickening of the skin in the palmar and plantar regions of the pastern [1–3]. Its multifactorial nature not only complicates the scientific investigation of its pathogenesis, but in clinical practice it also impedes the identification of the relevant underlying cause(s) and the formulation of appropriate treatment approaches.

A critical role is often attributed to bacterial infections and, accordingly, antimicrobial treatment of this condition is regularly undertaken. Specifically, infection with *Staphylococcus aureus*, which is known for its opportunistic pathogenicity [4] and association with skin and soft tissue infections [5, 6], is frequently suspected in the context of EPD [1, 3, 7, 8]. *S. aureus* can be found in a proportion of the normal skin flora of humans, and can also colonize different animals, including horses [5]; yet, not appearing as a typical commensal on their skin [9, 10], but being found rather in the nasal cavities, if present [11, 12]. Furthermore, *S. aureus* is also a major opportunistic pathogen that causes a variety of infections in both humans and animals, with the nasal mucosa often serving as a reservoir for endogenous infection [13, 14]. The acquisition and expression of different virulence factors is presumed to play a role in skin and soft tissue infections, such as e.g. intercellular adhesins promoting biofilm formation [15], cytotoxins challenging many different cell types of the hematopoietic lineage [16], or superantigens excessively triggering the immune system such as the ESAT-6-like (early secreted antigen target 6 kDa) staphylococcal type VII secretion system known as ESAT-6 secretion system (ESS) [17, 18].

Furthermore, *S. aureus* has the ability to become resistant to antimicrobials by either the acquisition of specific genes on mobile genetic elements or by DNA mutations in target genes [19]. According to the degree of resistance, *S. aureus* has been categorized into methicillin-susceptible *S. aureus* (MSSA) and methicillin-resistant *S. aureus* (MRSA) [20]. MRSA are resistant to all beta-lactam antibiotics, except for anti-MRSA cephalosporins, through the acquisition of methicillin resistance genes (e.g. *mecA, mecC*) on the staphylococcal cassette chromosome *mec* (SCC*mec*) [21, 22]. MRSA are also frequently resistant to other classes of critical antibiotics such as aminoglycosides, macrolides and fluoroquinolones [23]. Although MSSA are generally susceptible to antimicrobials, some of them can also exhibit resistance to these classes of antimicrobials [20].

Yet, the involvement of *S. aureus* in EPD as a primary and/or secondary pathogenic factor has not been elucidated, prompting us to first investigate the prevalence of *S. aureus* colonization in affected vs. unaffected pasterns, and the nasal passages of the respective horses. We hypothesized that the frequency of colonization would be increased in pasterns affected by EPD, and that an association would exist between nasal and pastern colonization. Furthermore, we anticipated that the frequency of colonization would deviate depending on the clinical manifestation and on the pretreatment. Also, analysis of whole genome sequencing (WGS) data should give new insights into the genetic diversity, local dissemination, as well as the virulence factors and antimicrobial resistance determinants of *S. aureus* in horses with and without EPD.

## Results

### Study population

Two hundred horses were enrolled in this study, 105 affected by EPD and 95 unaffected (Table 1). Horses were of various breeds and their stables were spread widely across parts of Switzerland. The affected horses originated from 64 different stables. From 24 of these stables, unaffected control horses could be recruited (n = 58). In addition, another 37 control horses from 13 independent stables were sampled. Of the affected horses, 46 were assigned to the mild form of EPD, 32 to the exudative form, and 27 to the proliferative form. Altogether, an antimicrobial had been applied previously in 28 horses, disinfectants solutions or ointments in 46 horses, and 31 of the affected horses had not been treated at all (Table 1).

**Table 1:** Characteristics of the study population

	EPD-affected horses	Control horses
**Total number**	105	95
**Age (year)**	11.7 (± 5.6)	13.6 (± 5.9)
**Sex**		
stallion	17	4
gelding	45	51
mare	43	40
**EPD form**		
mild	46	0
exudative	32	0
proliferative	27	0
no signs of EPD	0	95
**EPD score of sampled pasterns**	10.2 (± 2.8)	0 (± 0)
**Pretreatment of sampled pasterns**		
none	31	95
disinfectant	46	0
antimicrobial	28	0

For the pastern sample, all pasterns of each horse were examined for signs of equine pastern dermatitis (EPD) and were assigned to one of three EPD forms [1], as well as an EPD score that ranged between 0 (not affected) to 21 (severely affected)

### Frequencies of detection of *S. aureus* in bacterial culture

**Fig. 1:**
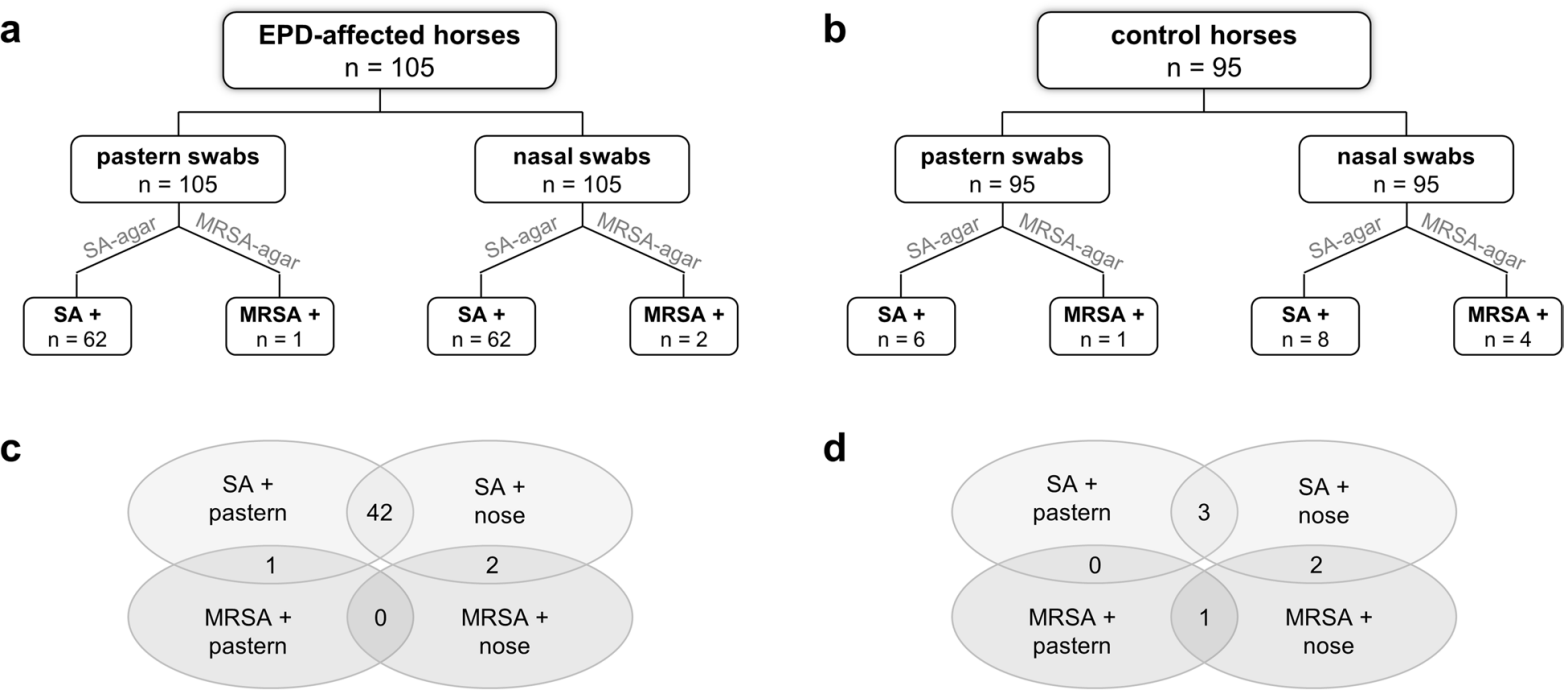
Overview of the investigated samples. The number of samples collected from (**a**) horses affected by equine pastern dermatitis (EPD) and (**b**) unaffected control horses is shown. Swabs were streaked onto two selective chromogenic plates for the selection of *Staphylococcus aureus* strains (SA+; MSSA and MRSA) and of methicillin-resistant *S. aureus* strains only (MRSA+). A modified form of a Venn diagram depicts that many (**c**) of the EPD-affected horses and (**d**) unaffected horses were simultaneously positive for *S. aureus* and/or MRSA in their noses and/or pasterns

In total, 127 *S. aureus* isolates were obtained from EPD-affected horses, and 19 isolates from healthy control horses (Fig. 1). Based on growth on both SaSelect and CHROMagar MRSA II plates, *S. aureus* was significantly more prevalent in swab samples from affected pasterns than from unaffected ones (affected: n = 62 [59.0 %], control: n = 6 [6.3 %], *P* < 0.001). The same was observed for the nasal swab samples (affected: n = 62 [59.0 %], control: n = 8 [8.4 %], *P* < 0.001). The frequency of MRSA positive samples was comparatively low and did not differ significantly between affected and control horses, neither in the pasterns (affected: n = 1 [0.9 %], control: n = 1 [1.1 %], *P* = 0.94) nor in the nose (affected: n = 2 [1.9 %], control: n = 4 [4.2 %], *P* = 0.34). A modified form of a Venn diagram depicts that many horses were simultaneously positive for *S. aureus* and/or MRSA in their noses and/or pasterns (Fig. 1).

In affected pasterns, the association between the EPD form at time of sampling and the choice for the type of pretreatment used was not significant in our study population (*P* = 0.43). Furthermore, the pretreatment was not found to play a predominant role in the probability of *S. aureus* isolation in affected pastern samples (*P*_*adj*_ = 0.54). However, the form of EPD showed a significant effect (*P*_*adj*_ = 0.03), with a higher ratio of positive cultures observed in exudative (23/32 [72 %]) and proliferative lesions (19/27 [70 %]) than in mild ones (20/46 [43 %]).

### Characterization and distribution of the *S. aureus* isolates based on WGS

In one control horse with its nasal swab being positive for both bacterial cultures (*S. aureus*- and MRSA-selective plate), sequencing results revealed that the respective strains were identical. This duplicate was therefore removed for the following analysis.

**Fig. 2:**
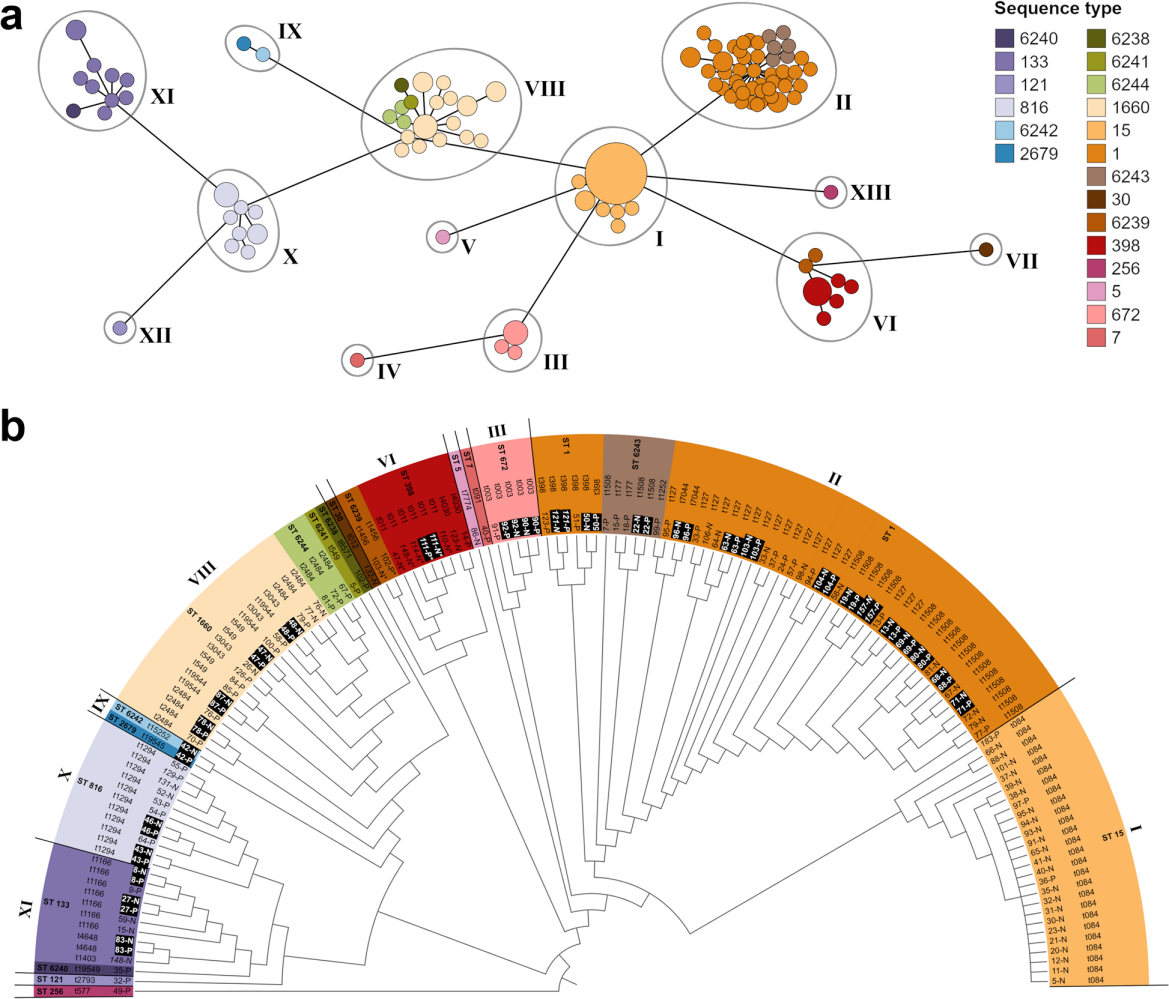
Genetic relationship between the isolates. (**a**) Minimum spanning tree of all investigated isolated, coloured by their sequence type (ST). The size of the nodes is proportional to the number of isolates represented by the respective node, as is the length of the branches to the number of allelic differences. Thirteen clusters (I-XIII) were gathered, adhering to a threshold of 4 or more matching alleles in MLST profiling. (**b**) Topological representation of genetic relationships of the investigated isolates. Sequence types are coded in the same colours as in (**a**). The branch lengths are relative and not to scale. Isolate IDs consist of the horse ID, followed by “P” for pastern samples or “N” for nasal samples. Isolates from control horses are written in italics. Methicillin-resistant strains are marked by an asterisk (*). Closely related isolates originating from the same horse are displayed with a black background

Altogether, 1549 genes were commonly present in all isolates and used for cgMLST analysis. In the generated cgMLST tree, 13 clusters or branches (I-XIII) of isolates were gathered, adhering to a threshold of 4 or more matching alleles in MLST profiling. There were six larger clusters of 10 to 51 isolates (clusters I, II, VI, VIII, X and XI), two smaller clusters of 2 to 5 isolates (clusters III and IX) and five singletons (branches IV, V, VII, XII and XIII). In total, 20 different ST were identified as well as 28 different *spa* types (Fig. 2).

The most abundant lineage was ST15-(*spa*)t084 (n = 26 [18 % of all isolates]) forming the whole of cluster I. Cluster II, the largest cluster, contained 51 *S. aureus* isolates including the second and third most abundant lineages ST1-t127 (n = 19 [13 %]) and ST1-t1508 (n = 18 [12 %]), as well as six isolates of ST6243 (a single *arcC* variant of ST1). Four of the other clusters (clusters VI, VIII, IX and XI) accommodated more than one ST, also generated by single locus variants namely ST398 and ST6239 (a single *pta* variant of ST398) in cluster VI, ST1660, ST6238 (a single *pta* variant of ST1660) and ST6244 (a single *glpF* variant of ST1660) in cluster VIII, ST2679 and ST6242 (a single *pta* variant of ST2679) in cluster IX, and ST133 and ST6240 (a single *aroE* variant of ST133) in cluster XI. The remaining clusters contained only one ST each or consisted of branches of singletons. Among the isolates of this study, 7 novel MLST patterns were detected, assigned as ST6238 to ST6244, as were 3 novel *spa* types, assigned as t19544, t19545 and t19549.

**Fig. 3:**
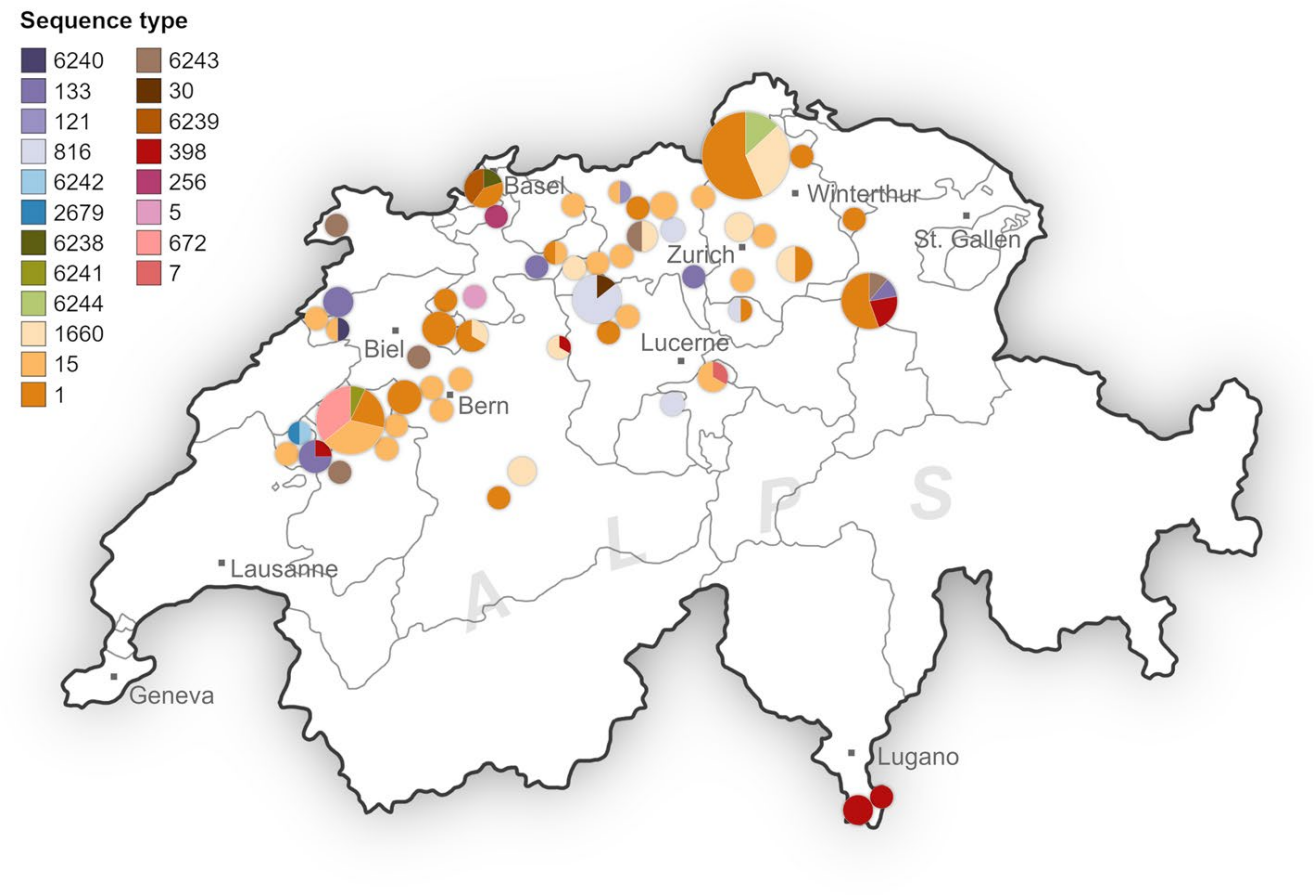
Distribution of sequence types. A map of Switzerland illustrating the distribution of sequence types within the visited stables. The size of the pie charts is proportional to the number of samples originating from the respective stable. It appears that cohabiting horses frequently harbour similar isolates. Map was drawn and illustrated by Sarah Kaiser-Thom

The observed strains displayed a moderately high degree of diversity, with half of the isolates belonging to either of two STs (ST1 or ST15). Furthermore, the horses often harboured highly related isolates in their nasal mucosa and on their pastern skin. In 46 horses, both the nose and skin swabs were positive for *S. aureus* (45 horses with MSSA and 1 with MRSA). Following molecular typing, 28 of these horses carried genetically related isolates of the same ST in both locations, 27 of which also matched in the *spa* type. Of note, *S. aureus* ST1 was almost exclusively associated with nasal carriage (Fig. 2). In addition, isolates of a same genotype were also frequently shared between horses of the same stable (Fig. 3).

The 8 MRSA strains were isolated from 7 different horses, and from either nose, pastern, or both. All MRSA gathered into the cluster VI with 6 isolates belonging to ST398-t011 and 2 belonging to ST6239-t1456. Notably, four of the six horses with MRSA ST398-t011 isolates lived in the same stable, just as the two horses with ST6239-t1456 isolates. All MRSA strains harboured the SCC*mec* type IVa.

While the *S. aureus* isolated from horses with EPD were represented in all clusters (I-XIII), the 18 isolates from healthy horses were distributed among 7 clusters (I, II, VI, VII, VIII, X, XI) associated with ST1-t084 (n = 1), ST15-t127 (n = 1), ST15-t398 (n = 3), ST15-t1508 (n = 2), ST398-t011 (n = 5), ST398-t14030 (n = 1), ST30-t012 (n = 1), ST1660-t3043 (n = 1), ST816-t1294 (n = 2), and ST133-t1403 (n = 1) (Fig. 2).

### Identification and distribution of antimicrobial resistance and virulence genes

Antimicrobial resistance genes were more abundant in MRSA than in MSSA. All MRSA (ST398 and ST6239) exhibited the same antimicrobial resistance profile harbouring the methicillin resistance gene *mecA*, the β-lactamase gene *blaZ*, the gentamicin, tobramycin, and kanamycin resistance genes *aacA-aphD*, and the tetracycline resistance gene *tet*(M). MRSA ST6239 had additional chromosomal mutations with amino acid substitutions within the fluoroquinolone resistance determining region of GyrA (S84L) and GrlA (S80F).

**Fig. 4:**
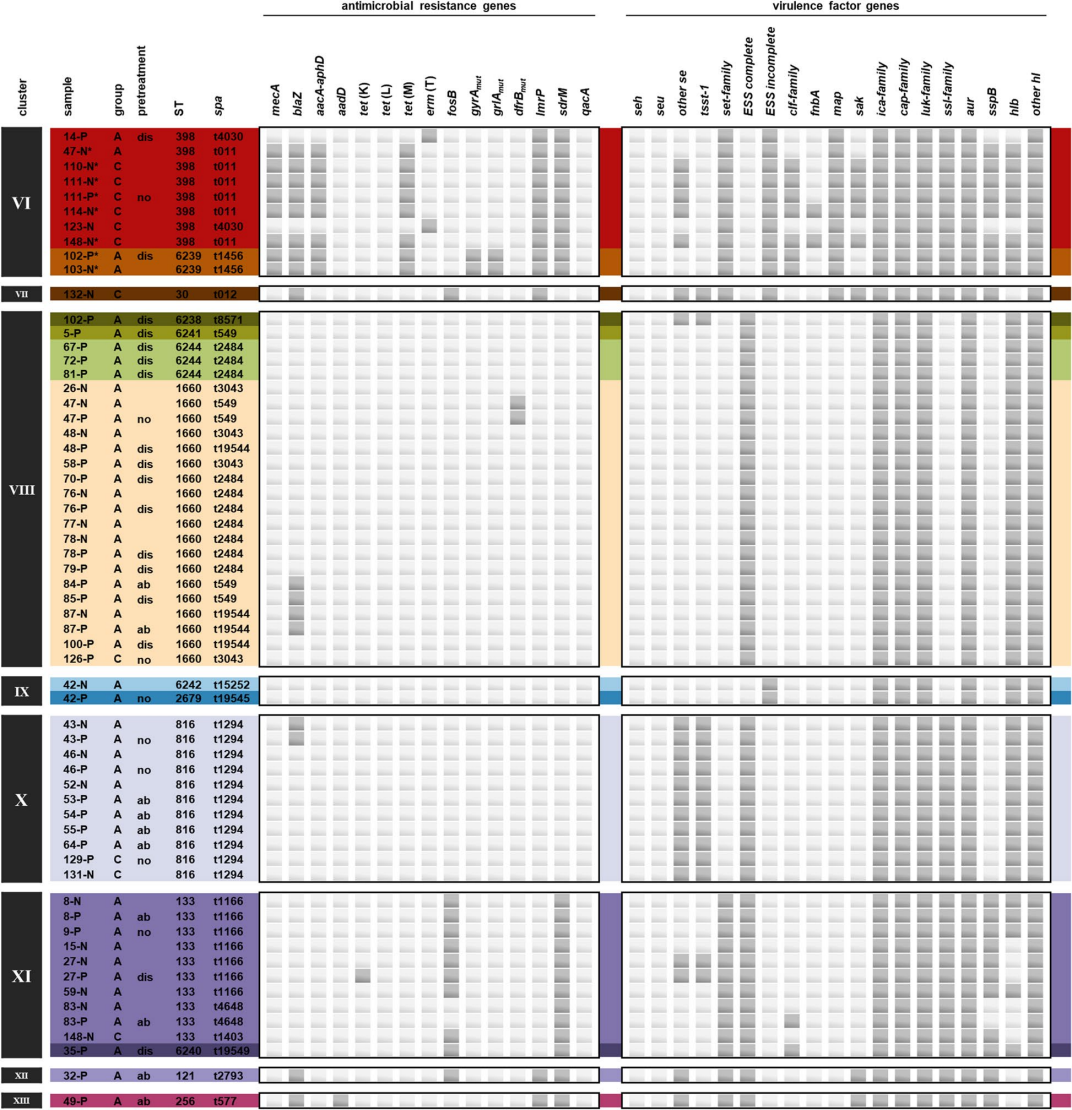
Distribution of detected antimicrobial resistance and virulence genes. Isolate IDs consist of the horse ID, followed by “P” for pastern samples or “N” for nasal samples. Key for column ‘group’: “A” = horse affected by EPD, “C” = control horse. In the column ‘pretreatment’, the type of pretreatment of the affected pasterns is disclosed: “no” = no antibacterial pretreatment, “dis” = disinfectant, “ab” = antibiotic. Further details on the clinical data can be found in the supplementary material. For a detailed key of the depicted genes please refer to the caption of Fig. 6

**Fig. 5:**
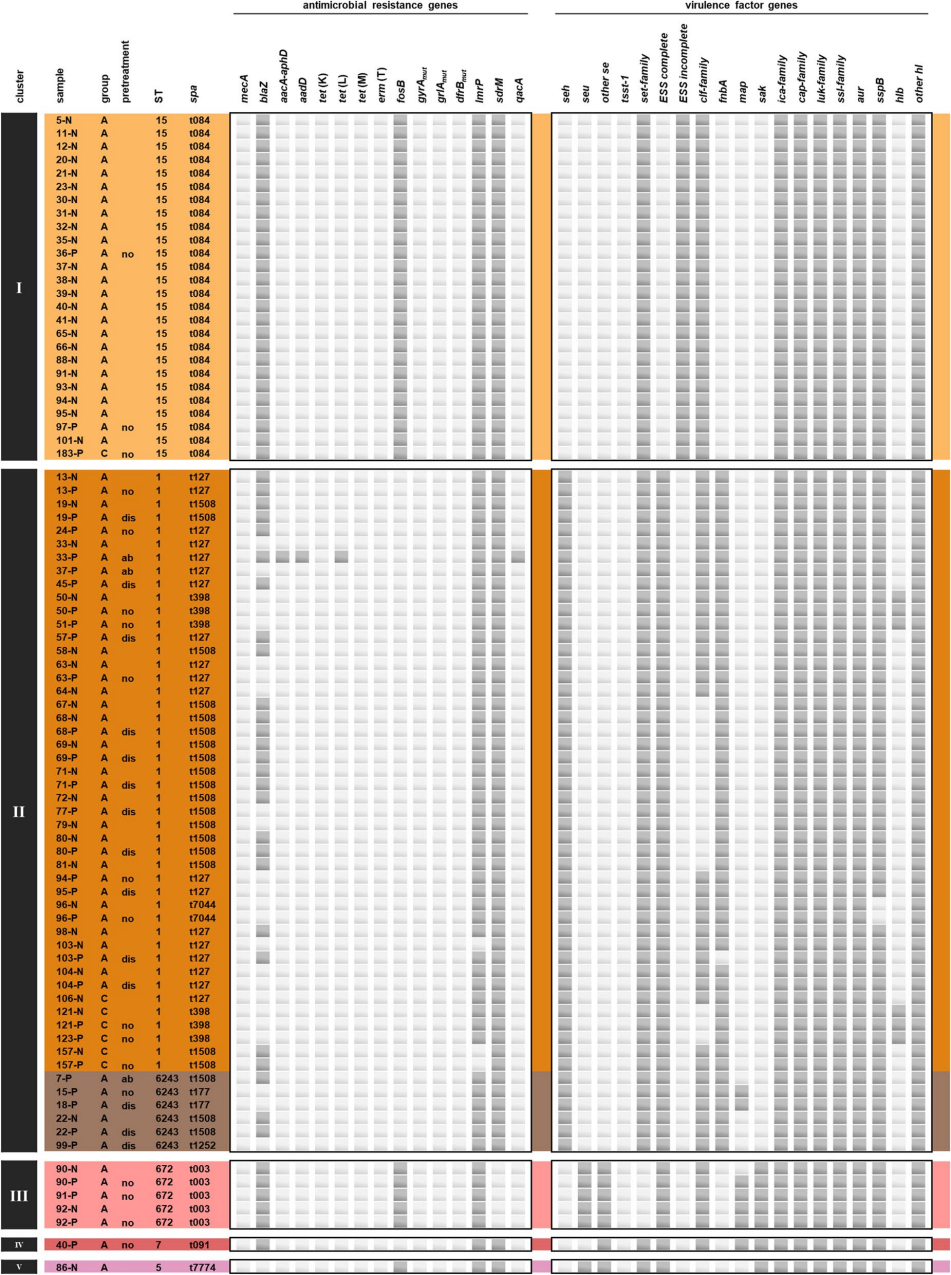
Distribution of detected antimicrobial resistance and virulence genes. Isolate IDs consist of the horse ID, followed by “P” for pastern samples or “N” for nasal samples. Key for column ‘group’: “A” = horse affected by EPD, “C” = control horse. In the column ‘pretreatment’, the type of pretreatment of the affected pasterns is disclosed: “no” = no antibacterial pretreatment, “dis” = disinfectant, “ab” = antibiotic. Further details on the clinical data can be found in the supplementary material. For a detailed key of the depicted genes please refer to the caption of Fig. 6

The antimicrobial resistance profiles of MSSA were mostly conserved within isolates of a same cluster. MSSA isolates contained zero to two known acquired resistance genes, except one isolate which had four. The *blaZ* and the fosfomycin resistance gene *fosB* were the most frequently detected acquired resistance genes in MSSA. Other genes, such as those associated with resistance to the aminoglycosides tobramycin and kanamycin (*aadD*), tetracyclines (*tet*(K), *tet*(L)), macrolides and lincosamides (*erm*(T)), as well as a mutation in the trimethoprim binding region of the chromosomal dihydrofolate reductase DfrB (F99Y) [24], were only sporadically found in single MSSA isolates. Furthermore, three acquired genes coding for different multidrug efflux pumps conferring resistance to various antibiotics and other antimicrobial agents (*lmrP*, *sdrM*, and *qacA*) [25] were found among both MRSA and MSSA (Figs. 4 and 5).

The isolate with the second highest frequency of AMR genes was an MSSA belonging to ST1, obtained from an EPD-affected pastern. The corresponding horse was one of two horses having the highest assigned EPD score in the clinical assessment (score: 17/21), and its medical history revealed prolonged treatment of EPD, including various antimicrobial ointments. Otherwise, no association between antimicrobial treatment of EPD and resistance profile could be made, since *S. aureus* exhibiting the same resistance pattern were found in both treated and non-treated horses. Further details on the clinical data as well as a breakdown of all antimicrobial resistance and virulence-associated genes can be found in the supplementary material (Additional file 1).

**Fig. 6:**
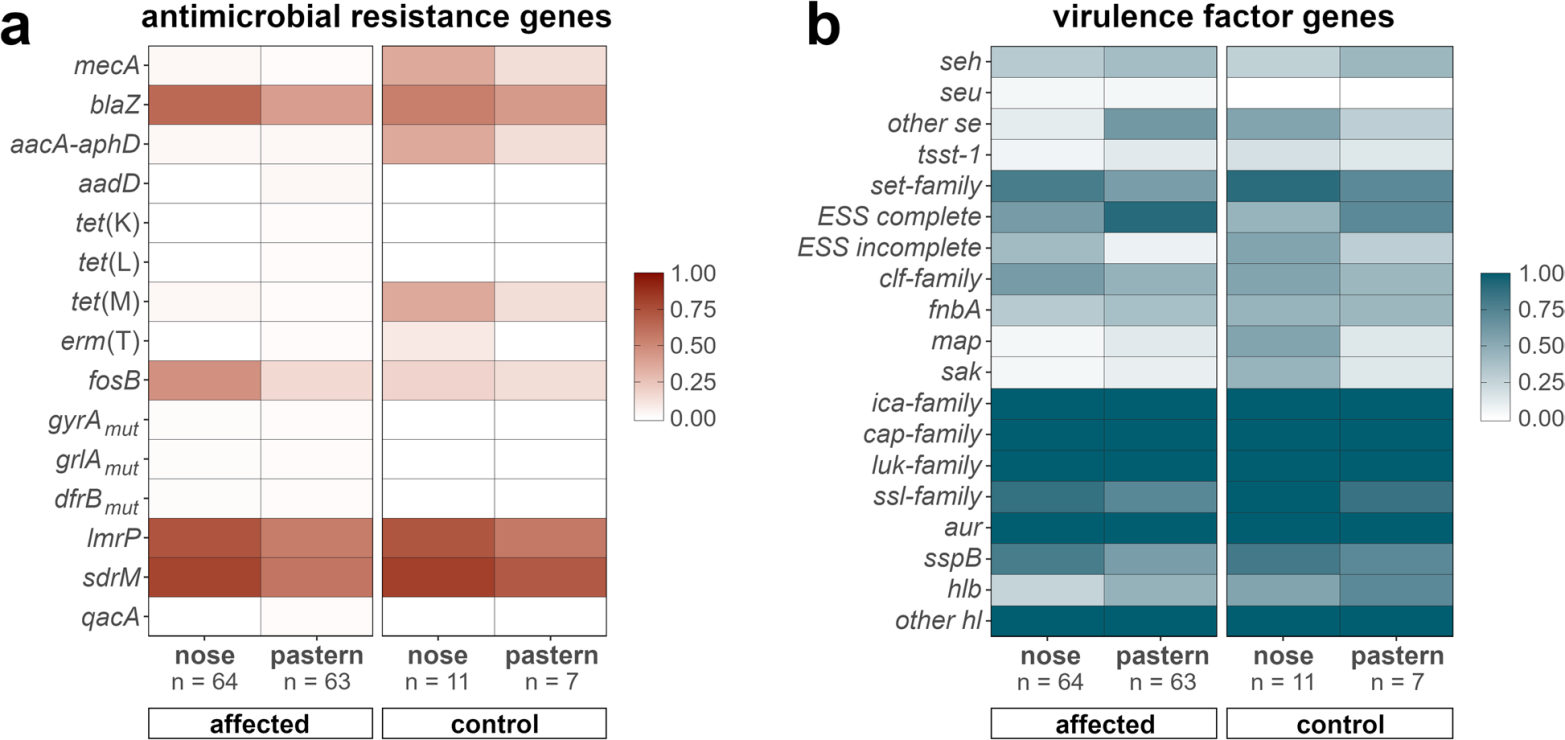
Heatmaps of detected antimicrobial resistance and virulence genes. Frequencies of selected antimicrobial resistance genes (**a**) and further virulence factor genes (**b**) in nose and pastern samples from horses affected by equine pastern dermatitis and unaffected control horses are depicted. Antimicrobial resistance genes and functions: MDT, *lmrP*, *sdrM* and *qacA*, multidrug transporters (MDT); *dfrB*_*mut*_, mutated chromosomal dihydrofolate reductase gene leading to amino acid substitution (F99Y) and trimethoprim (TMP) resistance; *gyrA*_*mut*_ (S84L) and *grlA*_*mut*_, chromosomal mutations with amino acid substitutions within the fluoroquinolone (FQ) resistance determining region of DNA GyrA (S84L) and topoisomerase GrlA (S80F); *fosB*, fosfomycin (FOS) thioltransferase gene; *erm*(T), macrolides, lincosamides and streptogramins B (MLS_B_) 23 S rRNA methylase gene; *mecA*, methicillin-resistance gene encoding PBP2a for resistance to all β-lactam-antibiotics; *blaZ*, β-lactamase gene; *tet*(K), *tet*(L), tetracycline (TET) efflux genes; *tet*(M), ribosome protection tetracycline resistance gene; *aacA-aphD*, aminoglycoside (AMG) acetyltransferase and phosphotransferase tandem genes for resistance to gentamicin, tobramycin and kanamycin; *aadD*, *ant(4’)-Ia*, amikacin and tobramycin nucleotidyltransferase gene. Virulence genes and their functions: *seh*, *seu*, and other staphylococcal enterotoxin genes (*sea*, *seb*, *sec*, *seg*, *sei*, *sel*, *sell*, *sem*, *sen*, *seo*, *sep*); *tsst-1*, toxic shock syndrome toxin-1 gene; *hlb* and other *hl* (*hld*, *hlgA*, *hlgB*, *hlgC*, *hlIII*, *hly*/*hla*); *ica*-family, genes associated with intercellular adhesion; *luk*-family, leucotoxin genes; *set*-family, genes for staphylococcal exotoxin-like proteins; “ESS complete” = all genes components of the ESAT-6 secretion system (ESS) present (*esaA*, *essA*, *essB*, *essC*, *esaB*, *esaG*, *esxA*, *esxB*, *esxC*, *esxD*, and *esaD*); “ESS incomplete” = five genes components of the ESS (namely *essC*, *esxB*, *esxC*, *esxD*, and *esaD*) missing; *clf*-family, fibrinogen-binding clumping factor genes; *fnbA*, fibronectin-binding protein gene; *map*, gene for immunomodulatory protein binding to extracellular matrix (ECM) components; *sak*, staphylokinase gene; *ica*-family, genes for intercellular adhesion; *cap*-family, genes for *S. aureus* capsular polysaccharides; *ssl*-family, genes for staphylococcal superantigen-like proteins; *sspB*, staphylococcal cysteine proteinase staphopain B gene

Overall, isolates from the nasal mucosa showed slightly higher frequencies of antimicrobial resistance genes than those from pastern samples, independently of being from healthy horses or horses affected by EPD (Fig. 6).

A total of 125 genes associated with bacterial virulence were identified in the genomes of the investigated *S. aureus* strains. Selected genes associated with toxins, superantigens, colonization, and immune evasion are illustrated in Figs. 4 and 5. The virulence factor gene profiles were highly conserved among *S. aureus* strains of a same cluster. Slightly different virulence profiles were observed within clusters II (ST15), VI (ST398), and XI (ST133), which further distinguished between strains displaying a different *spa* type within the same ST. The isolates possessed genes coding for proteins associated with intercellular adhesion (*ica*-family genes), capsule synthesis (*cap*), leukotoxins (*luk*), aureolysin (*aur*), and hemolysins (*hl* other than *hlb*). The *hlb* gene was found more frequently in pastern samples, as were staphylococcal enterotoxin genes (*se*), especially those that were not *seh*, and genes essential for the functionality of the ESS including those coding for the membrane proteins (*esaA*, *essA*, *essB* and *essC*), the cytosolic proteins (*esaB* and *esaG*), and the secreted substrates (*esxA*, *esxB*, *esxC*, *esxD*, *esaD*). In the respective isolates, the ESS was thus completed, while the system remained incomplete in the rest of the isolates, where *essC*, *esxBCD*, and *esaD* were missing. Genes of the ESS were lacking in isolates of the cluster I (ST1), VI (ST398) and IX (ST2679 and ST6242) which were almost exclusively composed of nasal isolates. On the other hand, genes coding for clumping factors (*clf*), staphylococcal superantigen-like proteins (*ssl*), staphopain B (*sspB*), and staphylococcal exotoxin-like proteins (*set*) were slightly more abundant in the nasal isolates (Fig. 6). Frequencies of AMR genes and virulence genes in EPD-affected horses and unaffected horses could not be statistically compared, as the respective groups of isolates differed considerably in size.

## Discussion

EPD is a multifactorial syndrome rather than a single disease entity and its complex pathogenesis is still not fully understood. Regarding bacterial infectious agents, staphylococcal species, particularly *S. aureus*, have frequently been implicated as primary or secondary pathogenic factors in the development of the disease [1, 3, 7, 8, 26–28]. The first objective of our study was, therefore, to investigate the prevalence of *S. aureus* colonization in pasterns of EPD-affected and unaffected horses, and to compare it to the frequency of respective isolates in the nasal mucosa, as the nasal passages represent a known reservoir for staphylococci [13, 29, 30]. Confirming our hypothesis, we found that the proportion of positive pastern samples in EPD-affected horses (59.0 %) was markedly higher than in unaffected horses (6.3 %). Opportunistic staphylococcal colonization of lesional sites has been described in various dermatological disorders and during wound healing in horses [27, 31]. However, it is important to note that *S. aureus* is not a typical commensal of the normal equine skin, unlike for example *S. sciuri* and *S. epidermidis* [9, 32]. The relatively low frequency of detection of *S. aureus* in unaffected horses is in accordance with these previous reports. Recently, our group investigated the skin microbiota in horse pasterns, where some of the here enrolled EPD-affected horses were included [33]. We found that, although *Staphylococcaceae* (microbiota composition was only resolved to the family level) were also detected in the unaffected pasterns, there was a substantial increase in relative abundance in the affected pasterns, particularly in exudative and proliferative lesions, essentially showing the same proportional pattern as we observed in the present study. As stated by Chiers et al., the isolation of pathogenic *S. aureus* from equine skin lesions, considering their relatively rare occurrence on healthy equine skin, suggests a role in the aetiology and progression of the respective lesions [27]. Our results thus indicate that *S. aureus* may also play a key role in EPD.

Of note, the affected horses concurrently exhibited a significantly higher number of *S. aureus*-positive nasal samples (59 %) as compared to the healthy control group (8.4 %), closely reflecting the numbers observed in the skin samples. The high rates of *S. aureus* isolation from both pasterns and noses of affected horses, coupled with the fact that the respective isolates were frequently also genetically highly related may indicate endogenous infection [13, 34]. Nevertheless, the cumulative occurrence of similar strains within stables also suggests horizontal transmission between horses, which could well extend to humans in contact with these horses, as has been reported before [12, 35]. Across all isolates, we observed only a moderately high diversity of STs and *spa* types, most of them already associated with *S. aureus* from horses (ST1-t127, ST1660-t549/t3043/t2484, ST15-t084, ST133-t1166, ST398-t011) [36–39], as well as from humans (ST15-t084, ST1-t127, ST1660-t549, ST1660-t3043, ST672-t003) [38–41]. Among them, ST15-t084, ST1-t127 and ST1-t1508 were the most predominant lineages. The lineage ST1-t127 seems to have a broad dissemination potential as it was found in diverse hosts, as for example in healthy pigs [42], healthy wild boars [43], nasal colonization and infections of humans [44], in humans in contact with pigs [45, 46], in mastitis of dairy cows [47], and in a purulent wound of a racehorse [48]. The reported strains frequently consisted of MRSA, contrarily to those of this study which were MSSA. The MRSA isolates in our study belonged either to the lineage ST398-t011 or to the novel, but closely related ST6239-t1456, and were all of SCC*mec* type IVa. MRSA of the clonal complex 398 represents the predominant lineage in livestock as well as in equine environments, being associated with both colonization and infections in horses [38, 49–51]. In our study, MRSA ST398 was more frequently detected in the nasal cavities of healthy horses than in the pastern of horses with EPD, suggesting that it may not have the virulence factors necessary to develop into EPD. For instance, they do not contain a complete ESS, like the MSSA ST15 which were most exclusively found in nasal samples. These *S. aureus* isolates lack the secreted substrate genes *esxB*, *esxC*, *esxD*, *esaD* and the membrane protein gene *essC*, which encodes for a membrane-anchored ATPase that putatively assembles into a complex with the other membrane components (*essA*, *essB* and *esaA*) and is necessary to release the aforementioned substrates [17, 18]. However, a markedly greater proportion of pastern isolates possessed both the substrate genes and all the genes of the ESS necessary for the secretion of these virulence factors. The substrates of this secretion system have previously been associated with the modulation of apoptosis in staphylococcal intracellular infection [52], as well as the ability of abscess formation [53], and might therefore be of importance in the pathogenesis of EPD. The pastern isolates also exhibited greater abundances for the *hlb* gene and for a larger variety of staphylococcal enterotoxins. The *hlb* gene encodes for a hemolytic toxin with sphingomyelinase activity and has been shown to play a crucial role in the ability to colonize the skin [54]. Amongst other properties of the enterotoxins, these heat-stable exotoxins can induce cytokine production as well as T-lymphocyte proliferation [55, 56], and may, consequently, induce or exacerbate inflammation. Moreover, a full range of additional virulence factor genes was encountered, providing the isolates competitive advantage as well as pathogenic potential. The isolates contained *cap* and *ica* genes, assuring capsule synthesis and formation of biofilms [15, 57], as well as leucocidin genes *luk*, aureolysin gene *aur*, and an array of hemolysin genes *hl*-, additionally paving the way for a more mobile and invasive behaviour [58–60].

Most of the *S. aureus* isolates did not contain multiple antimicrobial resistance genes, with the exception of the few MRSA ST398-t011 and ST6239-t1456, and one MSSA ST1-t127. Overall, the β-lactamase gene *blaZ* and the fosfomycin resistance gene *fosB* were the most frequently detected genes. Yet, penicillins and fosfomycin do not seem to be common active ingredients used in topical treatment of EPD, for which aminoglycosides were predominantly used. Metadata investigation of the participating affected horses indicated that the antimicrobial ointments applied in the 13 antibiotically pretreated pasterns each contained an aminoglycoside, either neomycin (12/13) or gentamicin (1/13), most frequently in combination with thiostrepton (8/13) and once in combination with gramicidin. However, aminoglycoside resistance genes were only detected in 8 isolates which consist of the MRSA and the multidrug-resistant MSSA ST1-t127. With regard to these, at least in our cohort, commonly utilized topical antimicrobials, the current molecular-based resistance status of the investigated staphylococci can be considered noncritical. However, it may be possible that some of the isolates exhibit some resistance phenotypes for which no known acquired mechanisms has yet been reported and absent in the current databases. Therefore antimicrobial susceptibility testing should be performed to insure targeted treatment.

## Conclusions

Our study highlights the potential importance of *S. aureus* in the development and pathogenesis of EPD, revealing a substantial higher abundance of *S. aureus* in affected horses, as well as specific genetic features of the *S. aureus* population. To our knowledge, a comparably extensive whole genome sequencing approach of *S. aureus* strains has not yet been performed in the equine setting. Whole genome sequencing gave new insights into the genetic features of the investigated *S. aureus* strains, particularly resistance and virulence factors, and framed their genetic relationships. Our observations suggest that dissemination of strains takes place between different sites within the same horses, and that spreading also occurs between horses living in the same stable. Some *S. aureus* lineages containing specific virulence factors such as ESS, hemolysin and enterotoxins were more present in affected pastern than in nasal cavities. It remains open, whether *S. aureus* plays a rather primary or secondary role in the development of EPD, and what the clinical significance of these toxins is. Further controlled and longitudinal studies on the bacterial impact in the pathogenesis of EPD, as well as investigations on colonization with *S. aureus* strains in affected horses will be needed to identify the key virulence factors contributing to the pathogenesis of EPD. However, our study underlines that the presence of *S. aureus* in horses should not be neglected in the diagnostic, prevention and treatment of EPD following antimicrobial susceptibility testing.

## Methods

### Study design

This study was part of a superordinate project to investigate the role of dermal bacteria in EPD. Some of the EPD-affected horses were included in a previous study by our group regarding the skin microbiota in EPD [33]. Participants were recruited through announcements on social media platforms. Sampling started in April 2019 and was completed in August 2020. Whenever possible, healthy control horses were recruited from the same stables as the affected horses. To achieve comparable group sizes, further healthy horses from unrelated stables were also included.

All horses underwent a general physical examination, followed by thorough inspection of all four pasterns. Diagnosis of EPD was based on evidence of typical clinical signs and lesion severity was scored using a standardized scoring system [33]. The score accounts for skin pathologies that are commonly associated with EPD, including scales, crusts, ulceration and formation of skin folds [1–3]; the cumulative value of score for each pastern can range between 0 (not affected) and 21 (severely affected) [33]. Affected pasterns were also assigned one of three EPD forms (mild, exudative or proliferative) as described by Yu [1]. Horses were designated as controls, if they had not shown signs of EPD in the preceding two years. In EPD-affected horses, lesional pastern areas were included irrespective of local disinfecting or antimicrobial pretreatment, except that no topical treatment which could impair bacterial growth was applied at the day of sampling. However, no horses treated with systemic antimicrobials or hospitalized in the preceding six months were included in this study.

### Sample collection

Two swab samples were obtained from every participating horse, one from the pastern skin and one from the nasal mucosa. In the affected horses, the most severely affected pastern was chosen for sampling based on the cumulative lesion score. As hind legs are generally more susceptible to EPD, for comparison, the pastern sample from the healthy horses was obtained by stroking the pastern regions of both hind legs consecutively with the same swab. The nasal swab was obtained through the right nostril in all horses, in order to facilitate sampling by head-shy horses. Samples were collected using flocked swabs (Puritan Opti-Swab, Puritan Medical Products, Gulford, ME, US). For sampling of the skin, swabs were slightly moistened with sterile 0.9 % saline solution. Swabs were then transported in 1 mL of liquid Amies medium and processed on the same day.

### Cultivation and identification of strains

Swabs were placed into Mueller-Hinton broth containing 6.5% NaCl for overnight enrichment at 37 °C with shaking. A loopful of the cultures was then streaked onto chromogenic plates SaSelect (Bio-Rad, Hercules, CA, US) for the selection of *S. aureus*, and CHROMagar MRSA II (Becton and Dickinson Company, Franklin Lakes, NJ, US) for the selection of MRSA. The plates were incubated for 24 h at 37 °C under aerobic conditions. Colonies were identified by matrix-assisted laser desorption/ionisation time-of-flight mass spectrometry (MALDI-TOF MS) (Bruker Daltonics GmbH, Bremen, Germany), and sub-cultivated onto trypton soy agar plates containing 5 % sheep blood (TSA-S, Becton and Dickinson Company, Franklin Lakes, NJ, US). The *S. aureus* isolates were preserved by freezing in glycerol stocks at -80 °C.

A chi-square test of independence was used to compare the number of samples with cultures positive for *S. aureus* and/or MRSA between EPD-affected and healthy control horses, and within the affected horses, to compare the number of positive cultures between the three EPD forms, as well as the type of previous treatment. When indicated, a Bonferroni correction for multiple comparisons was performed. A *P*-value of <0.05 was considered as statistically significant.

### Whole genome sequencing

DNA for library preparation was extracted directly from bacterial colonies grown on TSA-S using enzymatic lysis by Proteinase–K in combination with mechanical disruption by glass beads (PowerBead, Qiagen, Hilden, Germany). Extracts were purified using the AMPure XP paramagnetic bead-based chemistry (Beckman Coulter, Brea, CA, US). Libraries were prepared with the Nextera DNA Flex Library Prep Kit (Illumina Inc., San Diego, CA, US) following the manufacturer’s instructions. Whole genome sequencing (2 × 150 bp paired-end) was performed on an Illumina MiSeq platform (Illumina Inc., San Diego, CA, US) at the Next Generation Sequencing Platform, Institute of Genetics, University of Bern.

### Analyses of WGS data

The WGS raw data was imported into the commercially available software SeqSphere+ (version 7, Ridom GmbH, Münster, Germany). The preprocessing tool Trimmomatic [61] was used for quality-based filtering and downsampling. A core genome multilocus sequence typing (cgMLST) scheme was created with 1,861 queried target genes, as previously described [62]. All genes which were present in all isolates were defined as the core genome and included for further analysis. A minimum spanning tree was constructed and visualized with the help of GrapeTree [63] and iTOL [64]. The software SeqSphere+ was also used for multilocus sequence typing (MLST), staphylococcal protein A (*spa*) typing, as well as for the prediction of antimicrobial resistances using NCBI’s AMRFinder, and of virulence factors using the Virulence Factor Database (VFDB). Screening for antimicrobial resistance chromosomal mutations using ResFinder 4.1, and staphylococcal cassette chromosome *mec* (SCC*mec*) typing using SCC*mec*Finder 1.2 were performed using tools and default settings of the Center for Genomic Epidemiology (http://www.genomicepidemiology.org/). A map of Switzerland was created using the free software GIMP (https://www.gimp.org) to illustrate the spread of sequence types (STs) within the visited stables. The open-source software R [65] was used to construct heatmaps of the frequencies of antimicrobial resistance (AMR) genes and further virulence factor genes.

## Supplementary Information

**Additional file 1.** Details of the investigated samples. Excel spreadsheet providing further details on the clinical data as well as a breakdown of all antimicrobial resistance and virulence-associated genes.

## Data Availability

All whole genome sequencing data has been deposited in the NCBI Sequence Read Archive under BioProject PRJNA692738. The dataset supporting the conclusions of this article is included within the additional file of this article.

## Abbreviations

AMR: antimicrobial resistance
cgMLST: core genome multilocus sequence typing
EPD: equine pastern dermatitis
ESS: ESAT-6-like staphylococcal type VII secretion system
MLST: multilocus sequence typing
MRSA: methicillin-resistant *Staphylococcus aureus*
MSSA: methicillin-susceptible *Staphylococcus aureus*
SCC*mec*: staphylococcal cassette chromosome *mec*
*spa* typing: typing based on the *S. aureus*-specific staphylococcal protein A (*spa*) gene
ST: sequence type
WGS: whole genome sequencing

## References

[1] Yu AA (2013). Equine pastern dermatitis. Vet Clin North Am Equine Pract.

[2] Scott DW, Miller WH Jr Pastern Dermatitis. In: Scott DW, Miller WH, eds. Equine Dermatology, 2nd ed. Maryland Heights: Saunders Elsevier 2011; 260–261.

[3] Marsella R (2019). Clinical approach to pastern dermatitis. Manual of Equine Dermatology.

[4] Tong SYC, Davis JS, Eichenberger E, Holland TL, Fowler VG (2015). *Staphylococcus aureus* infections: Epidemiology, pathophysiology, clinical manifestations, and management. Clin Microbiol Rev.

[5] Peton V, Le Loir Y (2014). *Staphylococcus aureus* in veterinary medicine. Infect Genet Evol.

[6] Poulakou G, Lagou S, Tsiodras S (2019). What’s new in the epidemiology of skin and soft tissue infections in 2018?. Curr Opin Infect Dis.

[7] Colles CM, Colles KM, Galpin JR (2010). Equine pastern dermatitis. Equine Vet Educ.

[8] Watson R (2017). Wet skin conditions: the scourge of the UK winter. Equine Health.

[9] Nagase N, Sasaki A, Yamashita K, Shimizu A, Wakita Y, Kitai S (2002). Isolation and species distribution of staphylococci from animal and human skin. J Vet Med Sci.

[10] Sangiorgio D, Hilty M, Kaiser-Thom S, Epper P, Ramseyer A, Overesch G (2021). The influence of clinical severity and antibiotic treatment on bacteriological culture and the microbiota of equine pastern dermatitis. Vet Dermatol.

[11] Busscher JF, Van Duijkeren E, Sloet Van Oldruitenborgh-Oosterbaan MM. The prevalence of methicillin-resistant staphylococci in healthy horses in the Netherlands. Vet Microbiol. 2006;113:131–6. 10.1016/j.vetmic.2005.10.028 16303264

[12] Weese JS (2004). Methicillin-resistant *Staphylococcus aureus* in horses and horse personnel. Vet Clin North Am Equine Pract.

[13] Wertheim HFL, Melles DC, Vos MC, Van Leeuwen W, Van Belkum A, Verbrugh HA (2005). The role of nasal carriage in *Staphylococcus aureus* infections. Lancet Infect Dis.

[14] Islam MZ, Espinosa-Gongora C, Damborg P, Sieber RN, Munk R, Husted L (2017). Horses in Denmark are a reservoir of diverse clones of methicillin-resistant and -susceptible *Staphylococcus aureus*. Front Microbiol.

[15] Cramton SE, Gerke C, Schnell NF, Nichols WW, Götz F (1999). The intercellular adhesion (ica) locus is present in *Staphylococcus aureus* and is required for biofilm formation. Infect Immun.

[16] Yoong P, Torres VJ (2013). The effects of *Staphylococcus aureus* leukotoxins on the host: Cell lysis and beyond. Curr Opin Microbiol.

[17] Anderson M, Aly KA, Chen YH, Missiakas D (2013). Secretion of atypical protein substrates by the ESAT-6 Secretion System of *Staphylococcus aureus*. Mol Microbiol.

[18] Mietrach N, Damián-Aparicio D, Mielich-Süss B, Lopez D, Geibel S (2020). Substrate interaction with the EssC coupling protein of the type VIIb secretion system. J Bacteriol.

[19] Lindsay JA, Staphylococci (2019). Evolving Genomes. Gram-Positive Pathog.

[20] Monaco M, Pimentel de Araujo F, Cruciani M, Coccia EM, Pantosti A (2017). Worldwide epidemiology and antibiotic resistance of *Staphylococcus aureus*. Curr Top Microbiol Immunol.

[21] Peacock SJ, Paterson GK (2015). Mechanisms of methicillin resistance in *Staphylococcus aureus*. Annu Rev Biochem.

[22] Lakhundi S, Zhang K (2018). Methicillin-resistant *Staphylococcus aureus*: Molecular characterization, evolution, and epidemiology. Clin Microbiol Rev.

[23] Watkins RR, Holubar M, David MZ (2019). Antimicrobial resistance in methicillin-resistant *Staphylococcus aureus* to newer antimicrobial agents. Antimicrob Agents Chemother.

[24] Fowler PW, Cole K, Gordon NC, Kearns AM, Llewelyn MJ, Peto TEA (2018). Robust Prediction of resistance to trimethoprim in *Staphylococcus aureus*. Cell Chem Biol..

[25] Schindler BD, Kaatz GW (2016). Multidrug efflux pumps of Gram-positive bacteria. Drug Resist Updat.

[26] Devriese LA, Nzuambe D, Godard C (1984). Identification and characteristics of staphylococci isolated from lesions and normal skin of horses. Vet Microbiol.

[27] Chiers K, Decostere A, Devriese LA, Haesebrouck F (2003). Bacteriological and mycological findings, and in vitro antibiotic sensitivity of pathogenic staphylococci in equine skin infections. Vet Rec.

[28] Knottenbelt D (2013). A frustrating condition - pastern dermatitis syndrome. Equine Heal.

[29] Kluytmans J, van Belkum A, Verbrugh H (1997). Nasal carriage of *Staphylococcus aureus*: epidemiology, underlying mechanisms, and associated risks. Clin Microbiol Rev.

[30] von Eiff C, Becker K, Machka K, Stammer H, Peters G (2001). Nasal carriage as a source of *Staphylococcus aureus* bacteremia. N Engl J Med.

[31] Kamus LJ, Theoret C, Costa MC (2018). Use of next generation sequencing to investigate the microbiota of experimentally induced wounds and the effect of bandaging in horses. PLoS One.

[32] Ross AA, Rodrigues Hoffmann A, Neufeld JD (2019). The skin microbiome of vertebrates. Microbiome.

[33] Kaiser-Thom S, Hilty M, Axiak S, Gerber V. The skin microbiota in equine pastern dermatitis: a case‐control study of horses in Switzerland. Vet Dermatol. 2021 Apr 8. doi: 10.1111/vde.12955. Epub ahead of print. 10.1111/vde.12955 PMC9290916 33830562

[34] Wertheim HFL, Vos MC, Ott A, Van Belkum A, Voss A, Kluytmans JAJW (2004). Risk and outcome of nosocomial *Staphylococcus aureus* bacteraemia in nasal carriers versus non-carriers. Lancet.

[35] Weese JS, Archambault M, Willey BM, Dick H, Hearn P, Kreiswirth BN (2005). Methicillin-resistant *Staphylococcus aureus* in horses and horse personnel, 2000-2002. Emerg Infect Dis.

[36] Dastmalchi Saei H, Safari E (2019). Methicillin resistance and clonal diversity of *Staphylococcus aureus* isolated from nasal samples of healthy horses in Iran. Ann Microbiol.

[37] Mama OM, Gómez P, Ruiz-Ripa L, Gómez-Sanz E, Zarazaga M, Torres C (2019). Antimicrobial resistance, virulence, and genetic lineages of staphylococci from horses destined for human consumption: High detection of *S. aureus* isolates of lineage ST1640 and those carrying the *lukPQ* gene. Animals.

[38] Sieber S, Gerber V, Jandova V, Rossano A, Evison JM, Perreten V (2011). Evolution of multidrug-resistant *Staphylococcus aureus* infections in horses and colonized personnel in an equine clinic between 2005 and 2010. Microb Drug Resist.

[39] Scholtzek H, Walther E, Stöckle K (2019). Molecular characterization of equine *Staphylococcus aureus* isolates exhibiting reduced oxacillin susceptibility. Toxins.

[40] Grinberg A, Biggs PJ, Zhang J, Ritchie S, Oneroa Z, O’Neill C (2017). Genomic epidemiology of methicillin-susceptible *Staphylococcus aureus* across colonisation and skin and soft tissue infection. J Infect.

[41] Vali L, Dashti AA, Mathew F, Udo EE (2017). Characterization of heterogeneous MRSA and MSSA with reduced susceptibility to chlorhexidine in Kuwaiti hospitals. Front Microbiol.

[42] Franco A, Hasman H, Iurescia M, Lorenzetti R, Stegger M, Pantosti A (2011). Molecular characterization of *spa* type t127, sequence type 1 methicillin-resistant *Staphylococcus aureus* from pigs. J Antimicrob Chemother.

[43] Porrero MC, Mentaberre G, Sánchez S, Fernández-Llario P, Gómez-Barrero S, Navarro-Gonzalez N (2013). Methicillin resistant *Staphylococcus aureus* (MRSA) carriage in different free-living wild animal species in Spain. Vet J.

[44] Earls MR, Kinnevey PM, Brennan GI, Lazaris A, Skally M, O’Connell B (2017). The recent emergence in hospitals of multidrug-resistant community-associated sequence type 1 and *spa* type t127 methicillin-resistant *Staphylococcus aureus* investigated by whole-genome sequencing: Implications for screening. PLoS One.

[45] Aspiroz C, Lozano C, Vindel A, Lasarte JJ, Zarazaga M, Torres C (2010). Skin lesion caused by ST398 and ST1 MRSA, Spain. Emerg Infect Dis.

[46] Lozano C, Aspiroz C, Lasarte JJ, Gómez-Sanz E, Zarazaga M, Torres C (2011). Dynamic of nasal colonization by methicillin-resistant *Staphylococcus aureus* ST398 and ST1 after mupirocin treatment in a family in close contact with pigs. Comp Immunol Microbiol Infect Dis.

[47] Pilla R, Castiglioni V, Gelain ME, Scanziani E, Lorenzi V, Anjum M (2013). Long-term study of MRSA ST1, t127 mastitis in a dairy cow. Vet Rec Case Reports.

[48] Sekizuka T, Niwa H, Kinoshita Y, Uchida-Fujii E, Inamine Y, Hashino M (2020). Identification of a *mecA*/*mecC*-positive MRSA ST1-t127 isolate from a racehorse in japan. J Antimicrob Chemother.

[49] Price LB, Stegger M, Hasman H, Aziz M, Larsen J, Andersen PS (2012). *Staphylococcus aureus* CC398: Host adaptation and emergence of methicillin resistance in livestock. MBio.

[50] Huber H, Koller S, Giezendanner N, Stephan R, Zweifel C (2010). Prevalence and characteristics of meticillin-resistant *Staphylococcus aureus* in humans in contact with farm animals, in livestock, and in food of animal origin, Switzerland, 2009. Eurosurveillance..

[51] Abdelbary MMH, Wittenberg A, Cuny C, Layer F, Kurt K, Wieler LH (2014). Phylogenetic Analysis of *Staphylococcus aureus* CC398 reveals a sub-lineage epidemiologically associated with infections in horses. PLoS One.

[52] Korea CG, Balsamo G, Pezzicoli A, Merakou C, Tavarini S, Bagnoli F (2014). Staphylococcal Esx proteins modulate apoptosis and release of intracellular *Staphylococcus aureus* during infection in epithelial cells. Infect Immun.

[53] Burts ML, Williams WA, DeBord K, Missiakas DM (2005). EsxA and EsxB are secreted by an ESAT-6-like system that is required for the pathogenesis of *Staphylococcus aureus* infections. Proc Natl Acad Sci U S A.

[54] Katayama Y, Baba T, Sekine M, Fukuda M, Hiramatsu K (2013). Beta-hemolysin promotes skin colonization by *Staphylococcus aureus*. J Bacteriol.

[55] Thomas D, Chou S, Dauwalder O, Lina G. Diversity in *Staphylococcus aureus* enterotoxins. Superantigens and Superallergens. In Marone G, editor. Superantigens and Superallergens. Chem Immunol Allergy. 2007;93:24-41. 10.1159/000100856 17369698

[56] Ortega E, Abriouel H, Lucas R, Gálvez A (2010). Multiple roles of *Staphylococcus aureus* enterotoxins: Pathogenicity, superantigenic activity, and correlation to antibiotic resistance. Toxins.

[57] Sau S, Bhasin N, Wann ER, Lee JC, Foster TJ, Lee CY (1997). The *Staphylococcus aureus* allelic genetic loci for serotype 5 and 8 capsule expression contain the type-specific genes flanked by common genes. Microbiology.

[58] Morinaga N, Kaihou Y, Noda M (2003). Purification, cloning and characterization of variant luke-lukd with strong leukocidal activity of staphylococcal bi-component leukotoxin family. Microbiol Immunol.

[59] Laarman AJ, Ruyken M, Malone CL, van Strijp JAG, Horswill AR, Rooijakkers SHM (2011). *Staphylococcus aureus* metalloprotease aureolysin cleaves complement C3 to mediate immune evasion. J Immunol.

[60] Vandenesch F, Lina G, Henry T (2012). *Staphylococcus aureus* hemolysins, bi-component leukocidins, and cytolytic peptides: a redundant arsenal of membrane-damaging virulence factors?. Front Cell Infect Microbiol.

[61] Bolger AM, Lohse M, Usadel B (2014). Trimmomatic: A flexible trimmer for Illumina sequence data. Bioinformatics.

[62] Leopold SR, Goering RV, Witten A, Harmsen D, Mellmann A (2014). Bacterial whole-genome sequencing revisited: Portable, scalable, and standardized analysis for typing and detection of virulence and antibiotic resistance genes. J Clin Microbiol.

[63] Zhou Z, Alikhan NF, Sergeant MJ, Luhmann N, Vaz C, Francisco AP (2018). GrapeTree: Visualization of core genomic relationships among 100,000 bacterial pathogens. Genome Res.

[64] Letunic I, Bork P (2019). Interactive Tree of Life (iTOL) v4: Recent updates and new developments. Nucleic Acids Res.

[65] R Core Team. R: A language and environment for statistical computing. R Foundation for Statistical Computing, Vienna, Austria. 2018. Available online at https://www.R-project.org/.

